# Analysis of chromosome karyotype and genome size in echiuran *Urechisunicinctus* Drasche, 1880 (Polychaeta, Urechidae)

**DOI:** 10.3897/CompCytogen.v13i1.31448

**Published:** 2019-03-13

**Authors:** Zhenkui Qin, Xueyu Li, Danwen Liu, Qing Wang, Li Lu, Zhifeng Zhang

**Affiliations:** 1 Ministry of Education Key Laboratory of Marine Genetics and Breeding, College of Marine Life Sciences, Ocean University of China, Qingdao 266003, China Ocean University of China Qingdao China

**Keywords:** *
Urechis unicinctus
*, karyotype, genome size, flow cytometry

## Abstract

Karyotype and genome size are two primary cytogenetic characteristics of species, which are of great significance to the study of cytogenetics, taxonomy, phylogenesis, evolution as well as molecular biology. However, this basic cytogenetic information in echiurans is lacking. Therefore, we analyzed characteristics of karyotype and genome size in the echiuran worm *Urechisunicinctus* Drasche, 1880. In this study, coelomic cells of *U.unicinctus* were used for analyzing the genome size by a flow cytometry with chicken erythrocytes as DNA standard, and the 2C DNA content was determined to be 1.85 pg, which was corresponded to the genome size of 904.58 Mbp approximately. Furthermore, trochophores of *U.unicinctus* were dissociated and cells were utilized for preparing the chromosomes stained with DAPI, and the karyotype was determined as 2n = 30 (10m + 6sm + 6st + 8t), FN=52. Our data provided the basic cytogenetic information of *U.unicinctus*, which could be utilized in taxonomic study and whole-genome sequencing in future.

## Introduction

Echiurans (spoon worms) are a group of marine worms which are unsegmented, coelomate, bilaterally symmetrical and soft-bodied (Goto 2016). Traditionally, echiurans have been excluded from Annelida because of their non-segmented characteristics (Fisher and MacGinitie 1928). Recently, based on molecular phylogenetic data (Struck et al. 2007, Wu et al. 2009, Struck et al. 2011, Weigert et al. 2014, Andrade et al. 2015, Goto 2016;) the species in Echiura have often been considered as a group of derived annelids that secondarily lost segmentation. Thus, a controversial issue of whether echiurans belong to Annelida or a separate Echiura phylum has emerged and this needs to be elucidated from different research scopes. Chromosome karyotype and genome size are two important cytogenetic characteristics and have been applied widely in taxonomic, phylogenetic and evolutionary studies (Ipucha et al. 2007, Kashmenskaya and Polyakov 2008, Leitão et al. 2010, Palomina et al. 2017). Regrettably, only very few studies about chromosomes in the echiuran worms are available (Griffin 1899, Lefevre 1907, Singhal and Dattagupta 1980), which were reported several decades ago, and no report was related to their genome size. Therefore, these basic cytogenetic characteristics of echiurans need to be revealed urgently.

Karyotype, including chromosome number and composition, could reflect the taxonomic relationship between species and be used as a tool to explore biological diversity (Dobigny et al. 2004, Leitão et al. 2010, Cioffi et al. 2012). Gallardo-Escárate and Del Río-Portilla (2007) analyzed the cytogenetical relationships of three abalone species *Haliotiscorrugata* W. Wood, 1828, *H.fulgens* Philippi, 1845, and *H.rufescens* Swainson, 1822 based on their chromosomal morphology, and proposed that *H.rufescens* and *H.corrugata* are cytogenetically more similar to each other than to *H.fulgens*. Ipucha et al. (2007) discussed the phylogenetic relationship by analyzing the karyotypes of seven species from Nereididae, and suggested the karyotypes are relatively similar and stable in nereidid species at the family level, while the main mechanism of chromosomal evolution could be pericentric inversions.

Genome size is the total DNA content within a single copy genome and is also referred to C-value, which is specific in every species and ranges from 0.02 pg (*Pratylenchuscoffeae* Zimmermann, 1898, a plantparasitic nematode) to 132.83 pg (*Protopterusaethiopicus* Heckel, 1851, a marbled lungfish) in animals (Gregory 2018). C-value estimation is important for genomic sequencing and analysis (Gregory 2005). Nevertheless, variation of genome size in different species is rarely used as a direct or single factor in evolution analysis due to the C-value paradox (the phenomenon that C-value is inconsistent with the complexity of biological structure or composition), which means the genome size among organisms were diverse and possessed no relationship to organismal complexity (Gregory 2010).

*Urechisunicinctus*, a commercial echiuran worm inhabiting the U-shaped burrows in the coastal mud flats, has unique roles in animal evolution, coastal sediment improvement and marine drug development (Liu et al. 2015). In this study, chromosome counting and composition analysis of *U.unicinctus* were carried out in the well-developed trochophore for the first time, and the genome size of *U.unicinctus* coelomic cells was also determined using flow cytometry. We aim to reveal the basic cytogenetic characteristics of *U.unicinctus*, which provide useful information for taxonomic and genomic studies.

## Material and methods

### Animals

*U.unicinctus* adults with 9.96 ± 0.42 cm in body length were purchased from an aquatic product market, which were collected from a coastal intertidal flat in Yantai, China.

### Chromosome preparation and karyotype analysis

Sampling

The mature sperms and oocytes were obtained by dissecting the nephridia of the healthy *U.unicinctus*, respectively. Artificial fertilization was conducted by mixing sperms and oocytes at a ratio of 10:1 in filter seawater (FSW), and then these fertilized eggs were cultivated until hatched in FSW (19.7 ± 0.3 °C, salinity 29 PSU, pH 8.29 ± 0.02). The hatched trochophores were collected using a 500 mesh sieve.

Chromosome preparation and karyotype analysis

Chromosomes of *U.unicinctus* trochophores were prepared as described by Earley (1975) with some modifications. The larvae were incubated in FSW containing 0.02% colchicine for 2.5 h at room temperature, and then transferred into Ca^2+^/Mg^2+^-free Dulbecco’s phosphate buffered saline (CMF-DPBS, 137 mM NaCl, 2.7 mM KCl, 8.1 mM Na_2_HPO_4_×7H_2_O, 1.1 mM KH_2_PO_4_) for 30 min with continuous mild shaking to obtain the dissociated larval cells. Successively, the cells were treated with 0.075 mol/L KCl for 30 min, collected by centrifuging at 1200 g for 5 min, and then fixed three times with cold Carnoy’s fixative (3 ethanol: 1 glacial acetic acid) for 15 min each. After centrifugation, the cells were re-suspended in 50% glacial acetic acid aqueous solution, and then dropped onto preheated clean glass slides at 56 °C, and air-dried. Finally, these samples were stained with 10 µg/ml 4’, 6-diamidino-2-phenylindole (DAPI) (Solarbio, China) for 15 min, and were examined and photographed under a Nikon Eclipse 80i fluorescence microscope (Nikon, Tokyo, Japan).

The chromosome lengths, chromosome relative lengths and arm ratios from well-formed chromosomes in metaphase were measured and calculated using MICRO-MEASURE 3.3 software. Data were presented as mean ± SEM (n = 5). Chromosomes were classified according to the description of Levan et al. (1964), and the homologous chromosome was assigned based on the similarities in length and centromere position using PHOTOSHOP CS6 software. The idiogram was constructed according to the arm ratio and relative length of the chromosomes. The karyotype was classified as described by Stebbins (1971).

### Estimation of DNA content

Preparation of single cell samples

Coelomic fluids from 11 healthy worms were collected by syringe puncturing *U.unicinctus* body wall, respectively, and three duplicate samples were obtained from each individual. The coelomic cells were pelleted by centrifugation at 1000 g (4 °C) for 5 min, washed three times with PBS (pH 7.2), and then resuspended with PBS (pH 7.2). The suspension was added dropwise to the precooled 70% ethanol and fixed overnight at 4 °C. The next day, cell samples were collected through a 50 μm nylon mesh filtration, adjusted to 5×10^5^ cells/ml, digested with 20 μg/μl RNase A for 10 min and then stained with 1 μg/μl propidium iodide (PI) for 30 min in the dark at room temperature.

Chicken erythrocytes (2C = 25 pg DNA) were used as an internal standard (Darzynkiewicz and Juan 1997). Fresh blood was acquired by puncturing the heart and mixed with 5% sodium citrate to prevent coagulation, and the subsequential processing procedures were as described in above.

Flow cytometric analysis

Twelve samples, including a chicken erythrocyte, a *U.unicinctus* coelomic cell, and ten mixed samples containing 500 μl erythrocytes and 500 μl coelomic cells, were analyzed using a Coulter Cytomics FC500-MPL flow cytometer (Beckman, California, USA) equipped with a 488 nm laser source to detect the DNA content, and the output was processed in the software FLOWJO 7.6.1. Coefficients of variation (CV) were adjusted below 5% to ensure the reliability. The DNA content of *U.unicinctus* was then calculated according to Doležel et al. (2007) using the equation: Y = N/M × X (Y means the 2C DNA content of *U.unicinctus*, N means the fluorescence mean values of *U.unicinctus* samples, M means the fluorescence mean values of internal standard (chicken erythrocytes), and X means the 2C DNA content of internal standard). The genome size of *U.unicinctus* was calculated using the equation: genome size (bp) = (0.978 × 10^9^) × DNA content (pg).

## Results

### Chromosome characteristics of *U.unicinctus*

**Figure 1.**
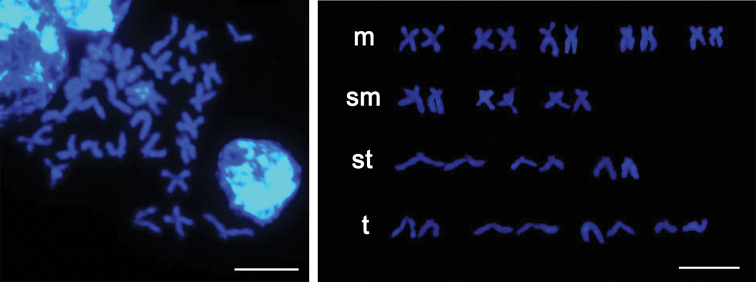
Metaphase chromosome and karyotype of *U.unicinctus* (2n = 30) stained with DAPI. m, metacentric chromosome; sm, submetacentric chromosome; st, subtelocentric chromosome; t, telocentric chromosome. Scale bar: 10 μm.

**Figure 2.**
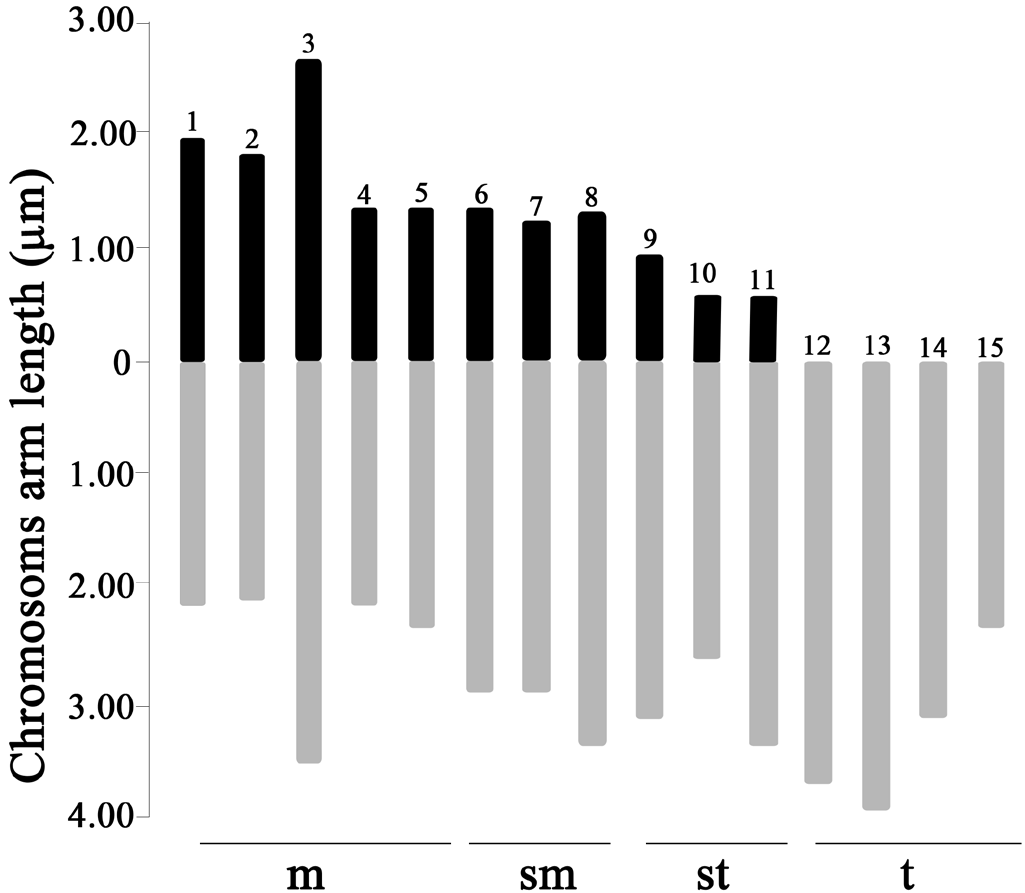
Chromosome idiograms of *U.unicinctus*. The dark regions showing short arms and the gray regions showing long arms.

The well dispersed metaphase chromosomes from *U.unicinctus* trochophore cells are shown in Fig. 1. The diploid chromosome number of *U.unicinctus* was 30, and fundamental number (FN) was 52. Fifteen pairs of the homologous chromosomes were matched, including 5 pairs of metacentric (m), 3 pairs of submetacentric (sm), 3 pairs of subtelocentric (st) and 4 pairs of telocentric chromosomes (t). No secondary constriction or satellite was found here. According to the measurement data (Table 1), the karyotype formula was deduced as 2n = 30 (10m + 6sm + 6st + 8t) (Fig. 1). Moreover, the idiogram was drawn based on the results above (Fig. 2). The index of karyotypic asymmetry (AsK) was 74.4%, which was the ratio of total length of long arms to that of all chromosomes. The chromosome size was 2.36–5.93 μm, with the longest to shortest chromosome length ratio (L/S) 2.51, and the percentage of chromosomes with arm ratio greater than 2:1 was 66.7%. Therefore, the karyotype of *U.unicinctus* was classified as 3B.

**Table 1.** Karyotypic parameters of *U.unicinctus* (2n = 30).

Chr	p (μm)	q (μm)	Total (μm)	RL (%)	AR	Type
1	1.97 ± 0.01	2.20 ± 0.02	4.17 ± 0.03	7.09 ±0.04	1.12 ± 0.01	m
2	1.82 ± 0.05	2.15 ± 0.06	3.97 ± 0.09	6.75 ±0.11	1.18 ± 0.03	m
3	2.45 ± 0.04	3.48 ± 0.01	5.93 ± 0.01	10.1 ±0.01	1.42 ± 0.01	m
4	1.42 ± 0.04	2.21 ± 0.02	3.63 ± 0.04	6.18 ±0.05	1.56 ± 0.05	m
5	1.43 ± 0.01	2.40 ± 0.02	3.82 ± 0.03	6.50 ±0.04	1.68 ± 0.01	m
6	1.42 ± 0.02	2.94 ± 0.05	4.36 ± 0.07	7.42 ±0.08	2.08 ± 0.02	sm
7	1.24 ± 0.02	2.94 ± 0.05	4.17 ± 0.06	7.09 ±0.07	2.37 ± 0.04	sm
8	1.29 ± 0.03	3.29 ± 0.09	4.58 ± 0.08	7.79 ±0.10	2.55 ± 0.10	sm
9	0.92 ± 0.02	3.08 ± 0.07	4.01 ± 0.09	6.82 ±0.11	3.35 ± 0.04	st
10	0.59 ± 0.02	2.59 ± 0.04	3.18 ± 0.06	5.41 ±0.07	4.40 ± 0.08	st
11	0.53 ± 0.01	3.29 ± 0.07	3.82 ± 0.08	6.50 ±0.10	6.27 ± 0.09	st
12	–	3.74 ± 0.08	3.74 ± 0.08	6.36 ±0.10	∞	t
13	–	3.95 ± 0.07	3.95 ± 0.07	6.72 ±0.08	∞	t
14	–	3.09 ± 0.02	3.09 ± 0.02	5.26 ±0.02	∞	t
15	–	2.36 ± 0.02	2.36 ± 0.02	4.01 ±0.02	∞	t

p, length of short arm; q, length of long arm; Total, total length of chromosome; RL, relative length of chromosome; AR, arm ratio of long arm to short arm from metaphase chromosomes. m, metacentric chromosome; sm, submetacentric chromosome; st, subtelocentric chromosome; t, telocentric chromosome.

### Genome size of *U.unicinctus*

**Figure 3.**
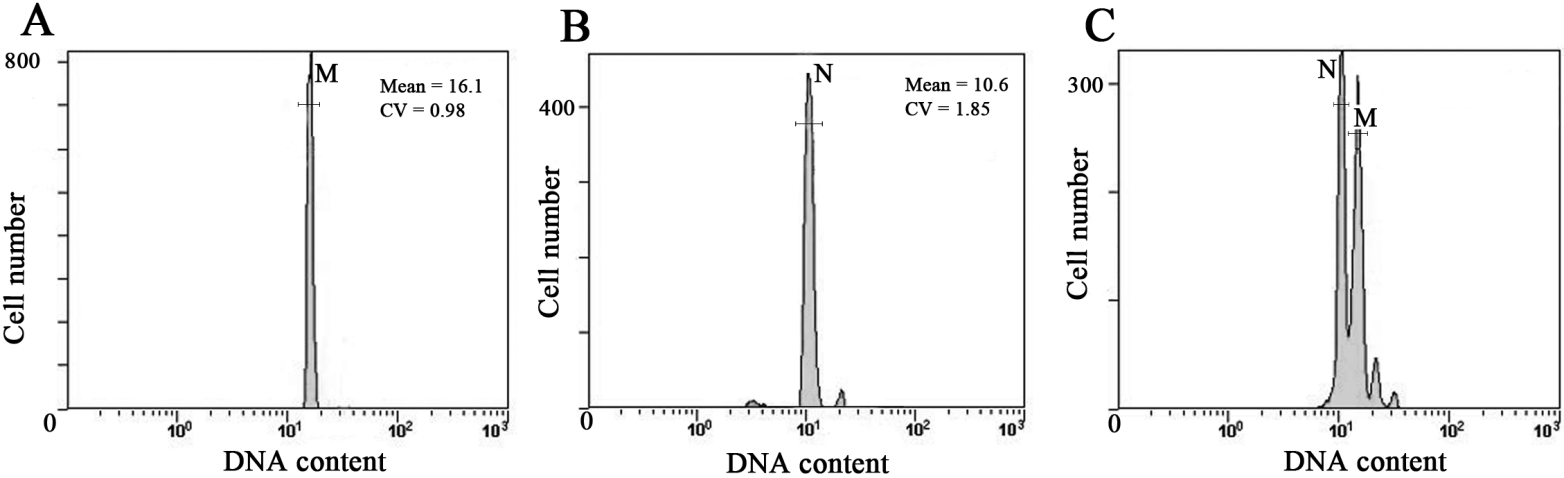
Estimation of nuclear DNA contents in *U.unicinctus* using flow cytometer. **A** Chicken erythrocytes **B***U.unicinctus* coelomic cells **C** one mixture sample of both. M, the 2C peak of chicken erythrocytes; N, the 2C peak *U.unicinctus* coelomic cells.

The frequency histograms of DNA content from chicken erythrocytes and *U.unicinctus* coelomic cells were presented based on the flow cytometric analyses (Fig. 3). No overlap between the two peaks (Fig. 3C) indicated that chicken erythrocytes as the internal standard was suitable for DNA content determination of *U.unicinctus* coelomic cells.

The 2C mean values of chicken erythrocytes (M) and *U.unicinctus* coelomic cells (N) in ten mixed samples and their ratios were presented in Table 2. The results showed that the average ratio of *U.unicinctus* coelomic cells to chicken erythrocytes was 0.74, therefore the 2C DNA content of *U.unicinctus* was calculated to be 1.85 pg, and its genome size was 904.58 Mb.

**Table 2.** Summary of DNA content and genome size of *U.unicinctus* estimated using flow cytometry.

**Sample**	**M**	**N**	**N/M**	**DNA content (pg)**	**Genome size (Mb)**
1	14.9	10.6	0.71	1.78	869.70
2	14.6	11.2	0.77	1.92	937.81
3	15.4	11.0	0.71	1.79	873.21
4	15.5	10.5	0.68	1.69	828.15
5	16.9	13.2	0.78	1.95	954.85
6	15.2	10.4	0.68	1.71	836.45
7	14.6	11.0	0.75	1.88	921.06
8	12.8	9.5	0.74	1.85	905.41
9	12.3	10.5	0.85	2.13	1043.60
10	14.8	10.6	0.72	1.79	875.57
Mean	14.7 ± 0.4	10.8 ± 0.3	0.74 ± 0.02	1.85 ± 0.04	904.58 20.21

## Discussion

Studies on the chromosomes in echiurans are very limited, and all of them were conducted several decades ago (Griffin 1899, Lefevre 1907, Singhal and Dattagupta 1980). The only one study of the karyotype was performed in a Bonellidae species, *Achaetobonelliamaculata* Fisher, 1953, (Singhal and Dattagupta 1980), which has 2n = 20 (10m), whereas others were focusing on the status and motion of chromosomes during cell division. Until now, no more karyotypic information of echiurans has been investigated. In this study, we obtained the clear karyotype of *U.unicinctus* using fluorescent staining technique and estimated its genome size as well. The karyotype of *U.unicinctus* was 2n = 30 (10m + 6sm + 6st + 8t), the 2C DNA content was 1.85 pg, and the genome size was 904.58 Mb approximately. This is the first study conducted in an Urechidae animal.

**Figure 4.**
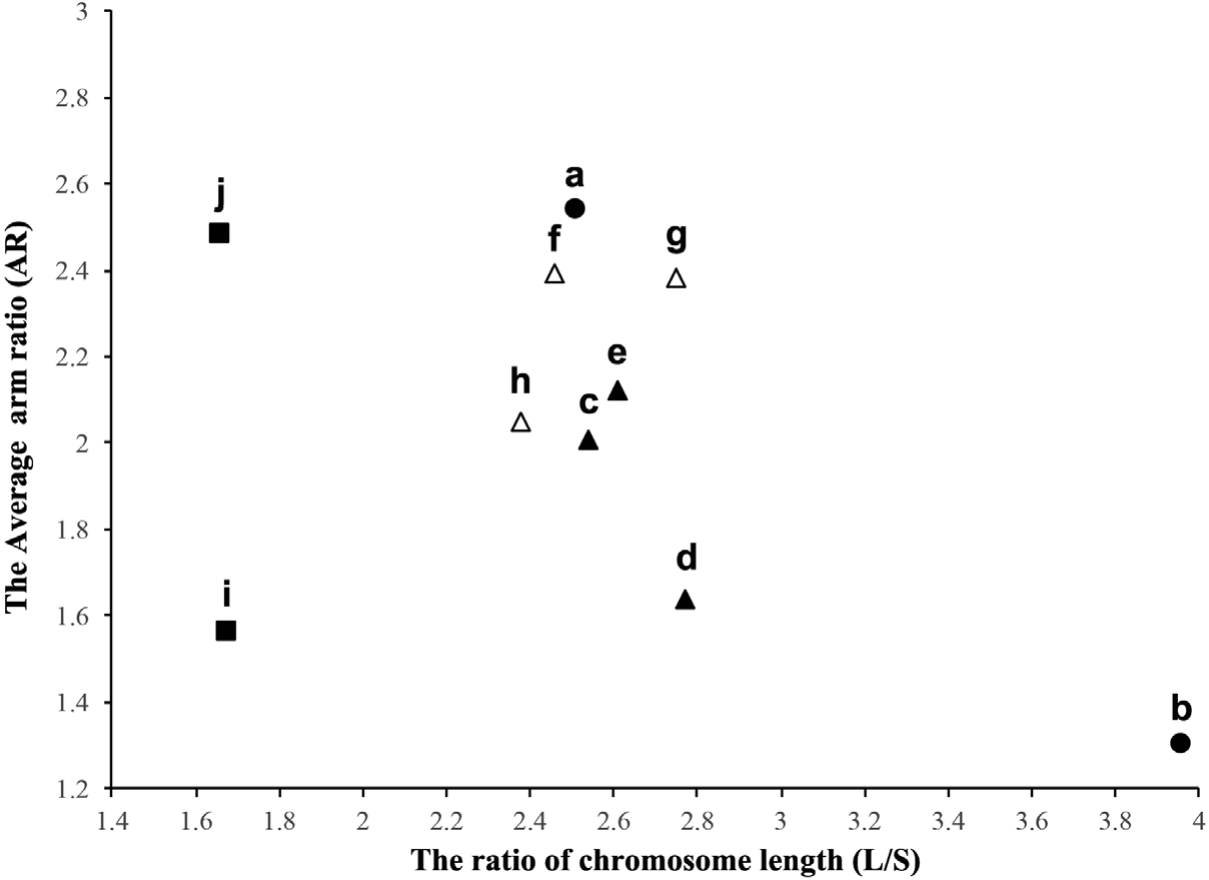
The karyotype asymmetry plot of echiurans, sipunculids and several annelids. **a** echiuran, *U.unicinctus***b** echiuran, *A.maculata***c** annelid, *N.oligohalina***d** annelid, *P.anderssoni***e** annelid, *H.diversicolor***f** annelid, *D.ghilarovi***g** annelid, *E.balatonica***h** annelid, *A.caliginosa***i** sipunculid, *S.nudus***j** sipunculids, *P.esculenta*. symbols: circle, echiurans; square, sipunculids; triangle, polychaeta; hollow triangle, oligochaeta.

Karyotypic information could be utilized to study the taxonomic relationships of species and biological diversity (Dobigny et al. 2004, Ipucha et al. 2007, Leitão et al. 2010, Cioffi et al. 2012). In this study, we collected the karyotypic data of multiple echiurans, sipunculids and annelids, and made some comparisons. It appeared that *U.unicinctus* possesses similar number and morphology of chromosomes with annelids (Table 3), and its karyotypic asymmetry was closely concentrated with annelids (Fig. 4). However, more data and analysis were required to determine the phylogenetic relationship between Echiurans and Annelids in future.

**Table 3.** The karyotypes of several echiurans, sipunculids and annelids species.

Species	Category	Karyotype	L/S^1^	AR^2^	Karyotype classification	Reference
*Urechisunicinctus* Drasche, 1880	Echiura	2n = 30 (10m + 6sm + 6st + 8t); FN = 52	2.51	2.54	3B	This study
*Achaetobonelliamaculata* Fisher, 1953	Echiura	2n = 20 (20m); FN = 40	3.96	1.03	1A	Singhal and Dattagupta 1980
*Sipunculusnudus* Linnaeus, 1766	Sipuncula	2n = 34 (26m + 8sm); FN = 68	1.68	1.56	2A	Wang et al. 2008
*Phasolosmaesculenta* Chen & Yeh, 1958	Sipuncula	2n = 20 (4m + 10sm + 6st); FN = 40	1.66	2.48	3A	Shi et al. 2013
*Nereisoligohalina* Rioja, 1946	Annelida: polychaeta	2n = 28 (14m + 2sm + 6st + 6t); FN = 50	2.54	2.01	4B	Ipucha et al. 2007
*Perinereisanderssoni* Kinberg, 1866	Annelida: polychaeta	2n = 38 (20m + 8sm); FN = 56	2.77	1.64	2B	Ipucha et al. 2007
*Hedistediversicolor* O.F. Müller, 1776	Annelida: polychaeta	2n = 28 (16m + 4sm + 8st); FN = 56	2.61	2.12	2B	Leitão et al. 2010
*Drawidaghilarovi* Gates, 1969	Annelida: oligochaeta	2n = 20 (6m + 8sm + 6st); FN = 48	2.46	2.39	3B	Anisimov et al. 2015
*Eiseniabalatonica* Pop, 1943	Annelida: oligochaeta	2n = 36 (10m + 20sm + 6st); FN =72	2.75	2.38	3B	Kashmenskaya and Polyakov 2008
*Aporrectodeacaliginosa* Savigny, 1826	Annelida: oligochaeta	2n = 36 (12m + 18sm + 6st); FN =72	2.38	2.05	2B	Kashmenskaya and Polyakov 2008

^1^ L/S: The ratio of longest to shortest chromosome length. ^2^ AR: The average arm ratio.

In general, genetic information of higher organisms is more complex than that of lower organisms, so the genomic size of higher organisms is relatively greater. However, there is no inevitable correlation between genome size and organismal complexity, because genome often contains a large number of highly repetitive DNA sequences, resulting in the conflict of DNA content and its evolutionary level. Gregory and Hebert estimated genome sizes from 12 species of freshwater oligochaetes ranging from 0.8 to 7.6 pg, and 15 species of earthworms varied from 0.4 to 1.2 pg (Gregory and Hebert 2002), suggesting that there is such a wide variation in the DNA content even between related species. Variation in genome size of polychaete taxa is not evenly distributed, as species inhabiting interstitial environments have smaller size (0.06–1.1 pg), whereas macrobenthic species are larger (0.4–7.2 pg), and the difference has been considered to adaptation of different environments (Soldi et al. 1994, Gambi et al. 1997, Gregory 2018). In addition, the DNA content among different species was also found to be independent of chromosome numbers, which was also concluded by EI-Shehawi and Elseehy (2017) that no correlation between genome size and chromosome number after the comparison of more than 6000 records. In echiurans, there has been no report of the nuclear genome size up to now. In the present study, the genome size of *U.unicinctus* was estimated as 1.85 pg, which is relatively small and could also be resulted from the adaptation to the hash and variable intertidal environment. The determination of *U.unicinctus* genome size maybe of little significance for the study of the evolutionary status of Echiura, but it could provide effective data support for large-scale whole-genome sequencing of *U.unicinctus* in the near future.

## Conclusion

In the present study, the karyotype of an Urechidae animal, *U.unicinctus*, was discovered for the first time as 2n = 30 (10m + 6sm + 6st + 8t), FN=52. Meanwhile, the 2C DNA content was detected to be 1.85 pg and its genome size was estimated as 904.58 Mb. Our study provided effective cytogenetic information for taxonomic study and whole-genome sequencing of *U.unicinctus*.
